# Supplementary material for: Assembly of Celastrol to Zeolitic Imidazolate Framework-8 by Coordination as a Novel Drug Delivery Strategy for Cancer Therapy
Source: Pharmaceuticals (Basel). 2022 Aug 29;15(9):1076. doi: 10.3390/ph15091076 (PMC9504028; doi:10.3390/ph15091076)
Supplement: Supplementary file 1 [file pharmaceuticals-15-01076-s001.zip › pharmaceuticals-1816213-supplementary.pdf]

Electronic Supplementary Information (ESI)

Assembly of celastrol to zeolitic imidazolate framework-8 as a new novel drug delivery strategy for cancer therapy

Na Wang[a†] and Yifan Li[a†], Fei He[a], Susu Liu[a], Yuan Liu[a], Jinting Peng[b], Jiahui Liu[c], Changyuan Yu*[a], Shihui Wang*[a]

[a] College of Life Science and Technology, Beijing University of Chemical Technology, Beijing 100029, China

[b] Department of Gynecology, Shenzhen Traditional Chinese Medicine Hospital, Shenzhen, 518033, Guangdong, China.

[c] Analytical Instrumentation Center, College of Chemistry and Molecular Engineering, Peking University, Beijing 100871, China.

Author for correspondence:

Tel: +86-10-64421335

Fax: +86-10-64421335

wangshihui@mail.buct.edu.cn

yucy@mail.buct.edu.cn

† These authors contributed equally to this work

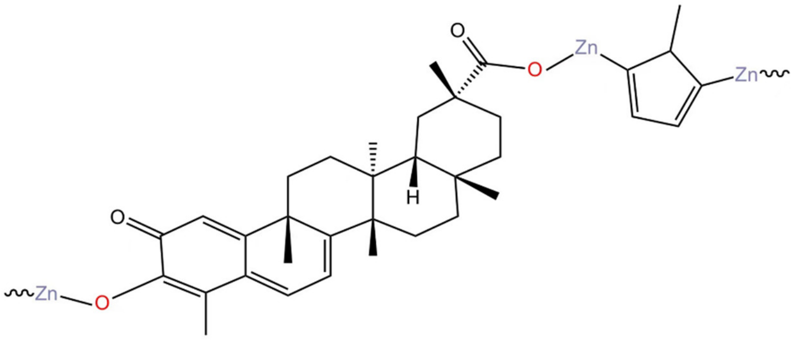

Fig. S1 The deduced chemical structure of Cel-ZIF-8.

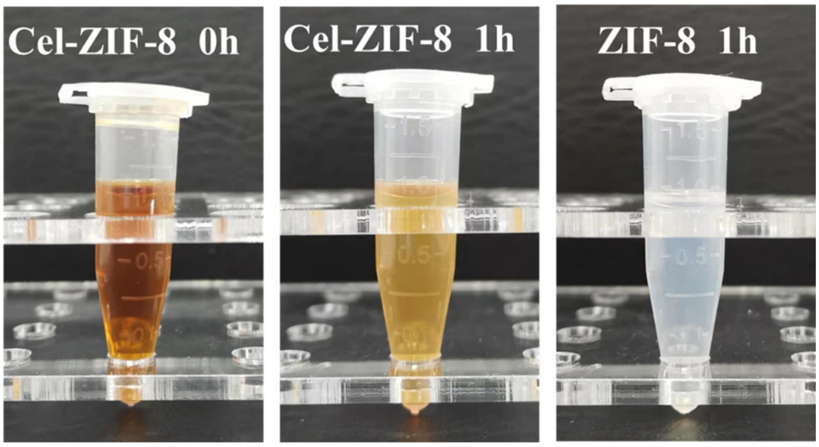

Fig. S2 The color changes of Cel-ZIF-8 during synthesis. ZIF-8 was used as the control.

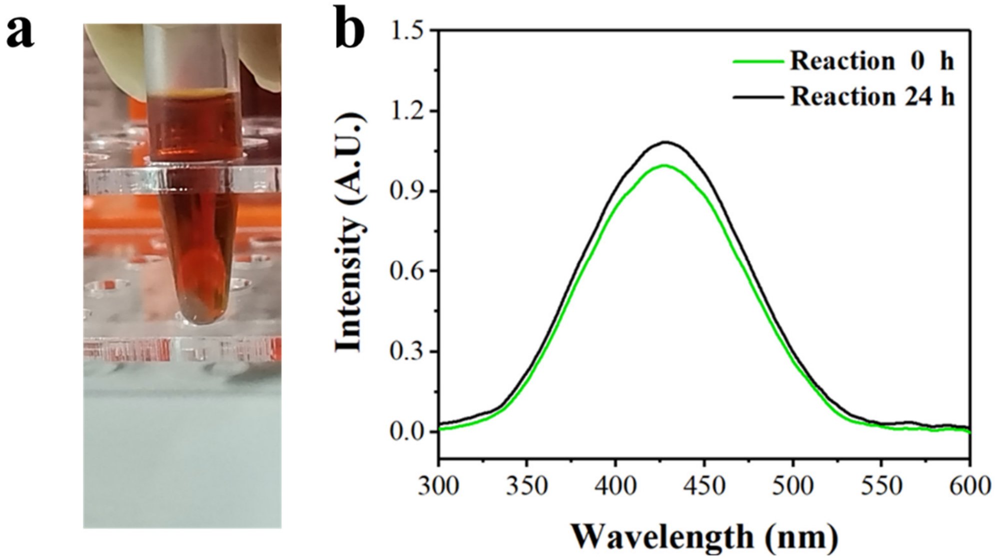

Fig. S3 Synthesis and Characterization of Cel/ZIF-8. Cel and ZIF-8 was mixed in methanol with stirring for 24 h and centrifuged at 10,000 rpm for 10 min (a). UV/Vis absorbance of the supernatant before and after stirring (diluted 20 times) (b).

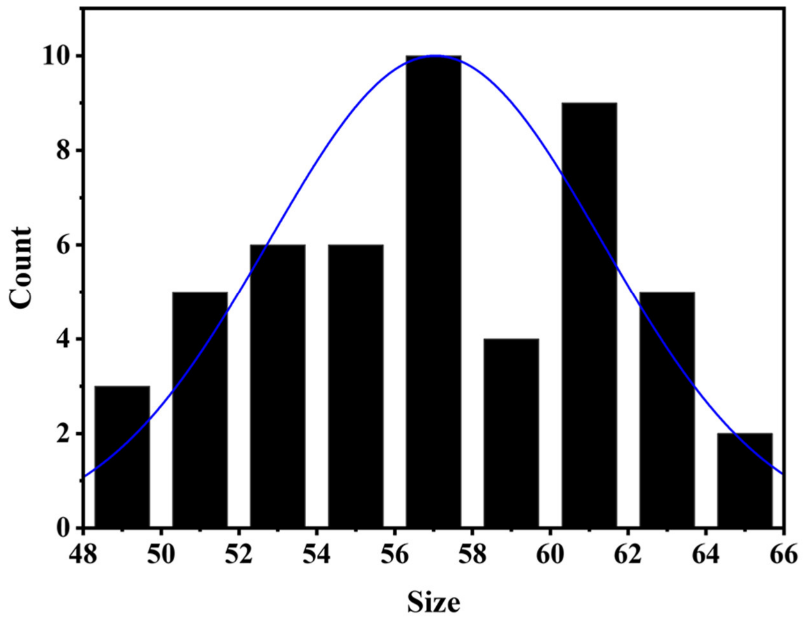

Fig. S4 Particle size distribution of Cel-ZIF-8 obtained from TEM image (57±9 nm).

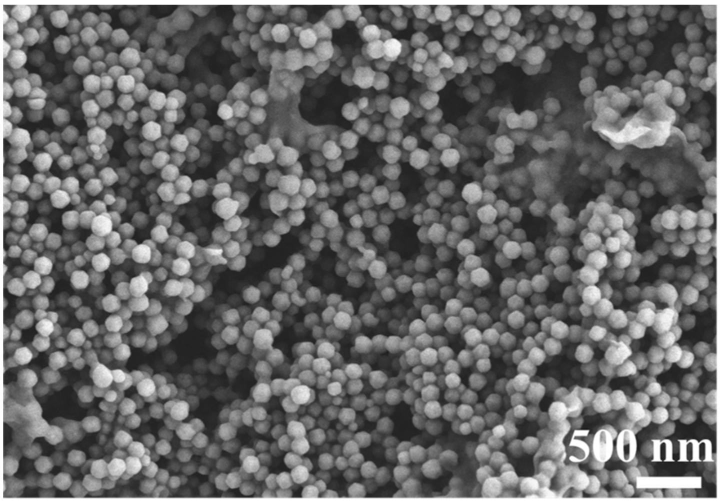

Fig. S5 The TEM image of ZIF-8 with an average size of 107±8.86 nm.

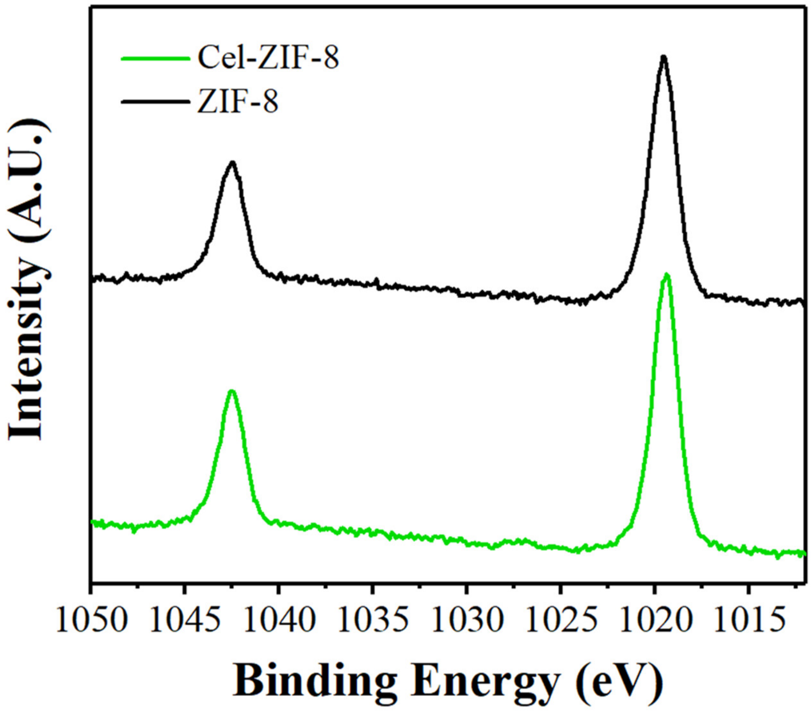

Fig. S6 Zn 2p of XPS of Cel-ZIF-8 and ZIF-8.

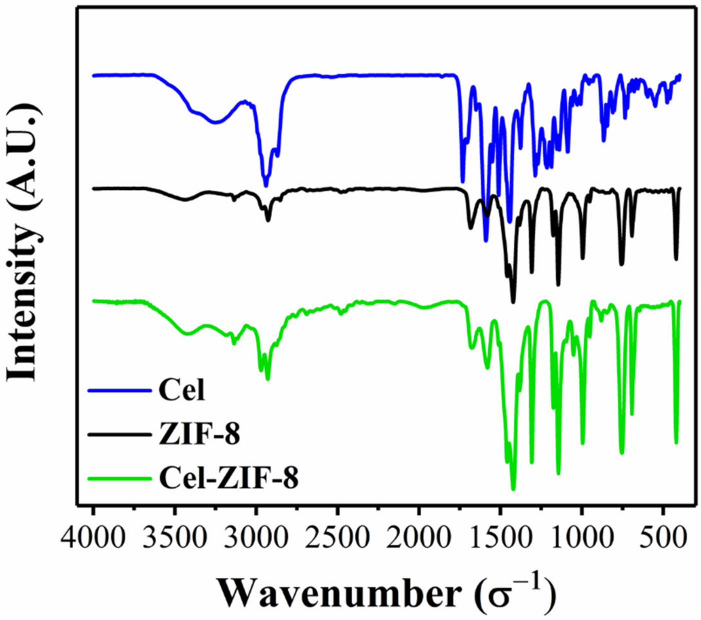

Fig. S7 FTIR spectra of Cel-ZIF-8, ZIF-8, and Cel.

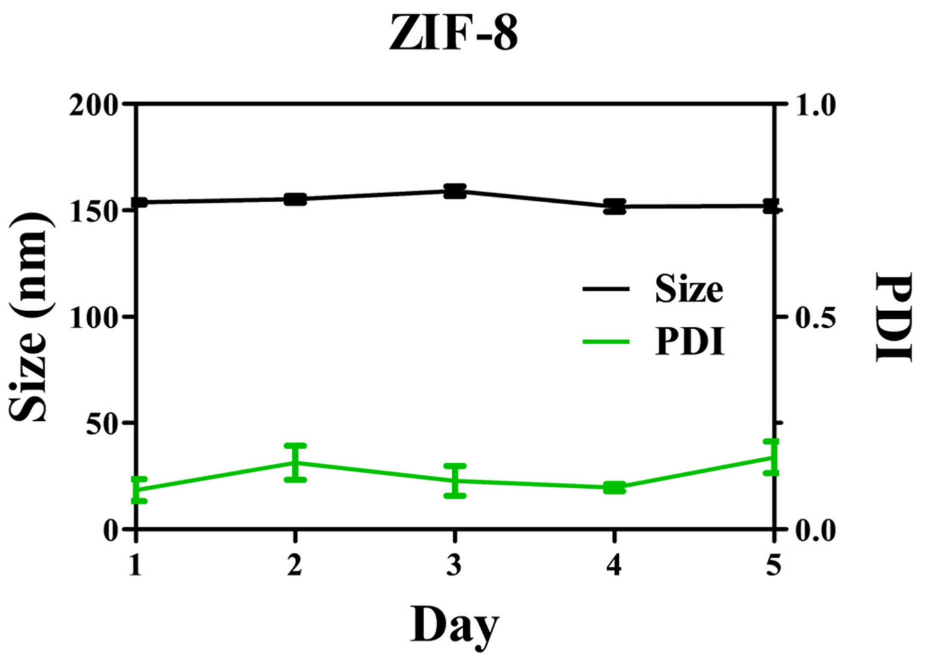

Fig. S8 Stability of ZIF-8 at physiological condition (pH 7.4).

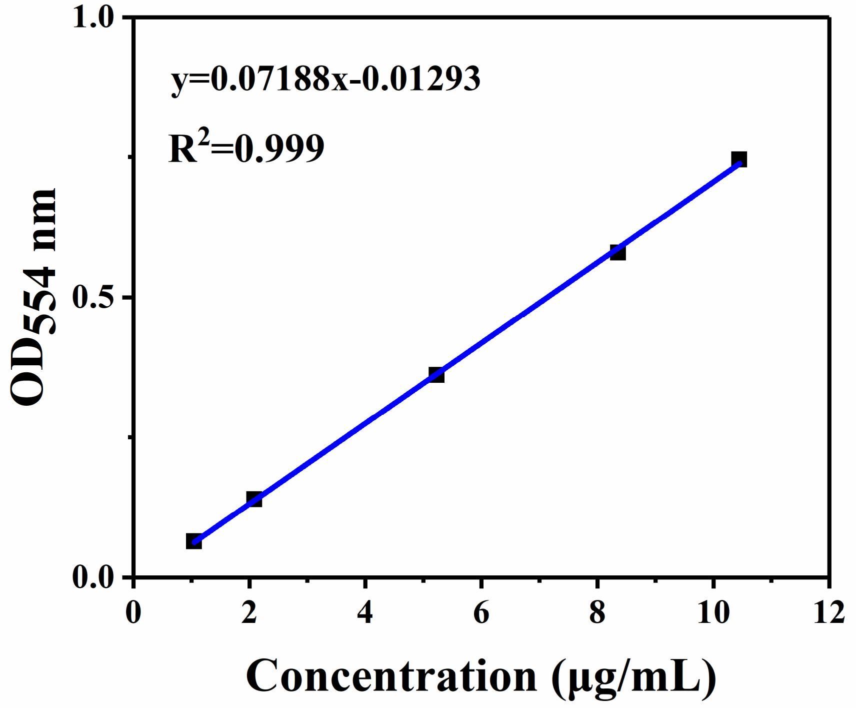

Fig. S9 Standard curve for Rhodamine B.
